# Human DNA collection from police dogs: technique and application

**DOI:** 10.1007/s12024-021-00355-3

**Published:** 2021-02-12

**Authors:** Alexandra Brower, Brice Akridge, Nancy Siemens-Bradley

**Affiliations:** 1 Department of Pathology and Population Medicine, Diagnostic Pathology Center, Midwestern University College of Veterinary Medicine, 5725 West Utopia Rd, Glendale, AZ 85308 USA; 2 Department of Public Safety, DNA Technical Unit, Central Regional Crime Laboratory, AZ Arizona Phoenix, USA

**Keywords:** DNA, Dog, Police, STR, Trace evidence

## Abstract

Police dogs are routinely deployed during criminal investigations under a variety of circumstances. In instances where police dogs are involved in apprehension of suspects, contact with a suspect may be observed or may occur out of the line of sight. The interactions between suspect and dog may include the dog biting the suspect, or the suspect touching or exuding bodily fluids onto the dog. In either form of contact, potentially valuable DNA may be left from the suspect on the dog. This paper describes a proof-of-concept study investigating collection of human DNA from the teeth and hair of dogs. It used controlled settings, where the human DNA sources were touch and saliva, and field cases, where the human DNA sources were unknown. The results of sample analysis to identify DNA short tandem repeats (STRs) from each of these scenarios are provided. They highlight the potential and importance of collecting trace DNA from police dogs who may have had contact with suspects during attempted apprehension.

## Introduction

The professed impetus behind training and using police dogs is to provide more effective law enforcement, aid in the capture of criminals, deter crime, and provide safety for officers. Law enforcement agencies appreciate that canines also add a source of persuasive, less-than-lethal force in suspect apprehension. A survey of all police and sheriff’s departments in California reported that officers responded to an average of 35 calls per month where it was expected a dog would be used. The most common uses identified were for searching areas or tracking suspects related to burglaries, robberies, and various felonies [1]. Officers deemed most of their calls to be high risk, and they employed the dog in the capture or interaction with suspects in most of these calls [1].

Canine deployments can be broken down into use as either weapons or tools [2, 3]. As weapons, the focus has been on the ability of dogs to attack and bite, and as tools, the focus has historically been on their excellent sense of smell. Recently however, with the expanded availability of body cameras, the tool role of police dogs has broadened to include gathering video that may be helpful in event reconstructions.

In this study we look at another expansion of the tool use of police dogs; the gathering of DNA evidence. In the course of apprehending a suspect, a police dog will most often come in contact with a suspect’s DNA through blood derived from a bite, but the suspect may also leave touch and other trace DNA on the dog, likely on the head as the dog is attempting a bite. While DNA profiles have been acquired from contact between individuals or objects for over two decades, identification of trace DNA evidence has greatly improved with STR PCR DNA profiling [4, 5]. This technology allows analysis of minute quantities of DNA and successful results are now obtained from even minimal contact [6, 7]. Below we describe the use of STR PCR DNA profiling in two technique development scenarios. In these controlled scenarios we attempt to collect and identify touch and saliva trace human DNA by swabbing canine hair and teeth. We then describe the use of swabbing to retrieve human DNA from the mouths of dogs involved in two police cases. The ability to collect trace human DNA from police dogs following contact with suspects adds yet another means by which police dogs can contribute to criminal investigations.

## Materials and methods

### Sample collection

Human buccal DNA swabs were self-collected by individuals designated as Control Persons A and B. Control Person A is the civilian owner of dogs used in a technique development scenario described below (scenario 1). Control Person B is the person who handles the dogs in scenario 1. These two individuals also supplied saliva DNA used in technique development, scenario 2, and they are the same individuals who collected postmortem samples from two field cases described below. These individuals supplied their DNA samples with informed consent.

Human DNA was collected from dogs as follows: samples collected for technique development, scenario 1 were swabbed from two dogs (Canines 1 and 2) by their owner, Control Person A. Samples collected for technique development, scenario 2 were swabbed from two police dogs (Canines 3 and 4), by their police officer handlers. Samples from dogs involved in police investigations (field cases 1 and 2) were collected by Control Persons A and B. All swabs used in this study were sterile, cotton-tipped, supplied in packages of two (Puritan), and are not considered forensic DNA grade. Two swabs were used at each collection site for all sampling scenarios.

All DNA processing and analysis was conducted in active crime laboratories, and samples were examined either as casework allowed or, in field case 2, as requested in the course of case adjudication. This resulted in variable hold times between collection and processing.

The following scenarios were designed as part of a proof-of-concept to determine if human DNA could be retrieved from canine hair or the surfaces of canine teeth in a quantity and quality that may be applicable to case investigations.

### Technique development, scenario 1

Control Person B handled the muzzle and rubbed the surfaces of the incisor and canine teeth of Canine 1. Within 2 min of this procedure, Control Person A donned gloves and, using sterile, dry, cotton swabs, rolled the swabs over the touched muzzle hair and outer surfaces of all four canine teeth of Canine 1. A similar procedure was followed for Canine 2, but in this case swabs were taken 15 min after the dogs muzzle and teeth were touched. The swabs were placed in pre-labeled, dry, cardboard boxes and stored at room temperature for 10 days prior to DNA extraction.

### Technique development, scenario 2

Approximately 3.0 cc of saliva was collected into glass tubes from Control Person’s A and B. Two canine unit police officers were provided a 5 min tutorial on how to swab the muzzle and mouth of their dogs. The saliva samples were then poured onto the gloved-fingers of the two canine police officers, and the officers in turn rubbed the surfaces of the incisor and upper canine teeth, and the dorsal surface of the muzzle of their police dogs (Canines 3 and 4). For Canine 4, the officer swabbed the muzzle using two sterile swabs, and then mouth, using an additional two swabs, all within 2 min of the saliva application. For Canine 3, the mouth and muzzle were swabbed within 2 min, and then again 15 min later by the dogs’ police handler. Swabs were stored in a similar manner to scenario 1 and DNA extraction was conducted 134 days after collection.

### Technique application, field case 1

Sterile swabs were used to collect DNA following the above procedure from the teeth of two dogs that had bitten a person. The time from the attack to collection of the samples was approximately 48 h. Swabs were stored as described above for 8 days prior to DNA extraction. All samples for scenario 1 and 2 and field case 1 were then submitted for processing following the Arizona Department of Public Safety Scientific Analysis Bureau protocols (AZDPS-DAP) [8].

### Technique application, field case 2

In this incident a suspect was bitten by a police dog during a criminal investigation. All four canine teeth and the incisor teeth were subsequently swabbed, processed, and analyzed for DNA using the above described procedure. The time lapse from the bite to collection of the sample was approximately 6 h. Swabs were stored as described above for 351 days prior to DNA extraction. All samples for field case 2 were then submitted for processing following the City of Phoenix Police Department Crime Laboratory DNA Analytical Protocol (PPD-DAP); copies are available by request to the Phoenix Police Department.

### DNA extraction

Samples were extracted using the Qiagen QIASymphony Investigator Kit and SP robotic platform using manufacturer recommendations and / or AZDPS-DAP/ PPD-DAP. Reactions were performed in 2.0 ml Qiagen Lyse&Spin® tubes. Incubation time of the swab heads in lysis buffer was for a minimum of 1 h to overnight at 56 °C shaking at 900 rpm. The elution volume was 100 µl. Samples for field case 2 were extracted as above, however standard 2.0 mL microcentrifuge tubes were used and the elution volume was 60 µl.

### DNA quantification

DNA quantification was performed using the Promega PowerQuant™ System with Life Technologies HID Real-Time PCR Analysis Software. Mastermix was prepared using 10.0 µl of 2X reaction mix, 1.0 µl of 20X primer/probe/IPC mix and 7.0 µl of amplification grade water per sample. Analysis of 2 µl samples was performed in a Life Technologies 7500 Real-Time PCR system according to manufacturer’s protocol and / or the AZDPS-DAP. DNA quantification by the PPD laboratory (field case 2) is as described above, with the Mastermix formulation being 10 µl of Quant Trio reaction mix and 8 µl of Quant Trio primer mix, and sample ratios per well of 8 µl Mastermix with 2 µl of sample per well.

### STR amplification

STR typing of all samples was accomplished using Promega PowerPlex®Fusion 6C (AZDPS Laboratory, scenarios 1 and 2 and field case 1), or Life Technologies GlobalFiler (PPD Laboratory, field case 2) PCR Amplification Kits. Promega PowerPlex®Fusion 6C reaction mix contained 5.0 µl of 5X Master Mix and 5.0 µl of 5X Primer Pair Mix per sample. Up to 15.0 µl sample containing a maximum of 500 pg DNA was added to the reaction mix. The Globalfiler reaction mix (PPD Laboratory) contained 7.5 µl of reaction mix, 2.5 µl of primer, and a 15 µl sample. PCR was performed according to a 29 cycle protocol on a Life Technologies model 9700 Thermalcycler. For all samples examined, a Life Technologies 3500 series Genetic Analyzer was used for capillary electrophoresis (POP-4, 36 cm array) following manufacturer’s recommendations and / or AZDPS or PPD-DAP. Data were analyzed with Life Technologies Genemapper® ID-X Version 1.5 software with analysis threshold of 60 rfu. Data generated per PPD-DAP were analyzed with Version 1.4 software.

## Results

The following results are summarized in Table 1, and sample data are provided as supplementary material. Partial human DNA profiles were identified in technique development, scenario 1, which involved control Person B touching the muzzle and teeth of Canines 1 and 2 with ungloved hands. A similar experiment using police dogs was conducted with saliva as the source of human DNA (technique development, scenario 2). When saliva was rubbed on the muzzle of Canines 3 and 4, human DNA collected from the dog’s hair was consistent with the control person who provided it. In this study the term consistent is used to describe a human DNA profile with one or more inconclusive loci. In scenario 2, teeth were also swabbed for DNA derived from human saliva, and in this instance DNA that matched the control person who provided it was found in samples taken within 2 min of the saliva sample being placed on the teeth, and 15 min later. In this study matched DNA indicates full human DNA profiles with no inconclusive loci. In field case 1, 48 h had passed before sample collection from the mouths of two dogs that had bitten a person was possible. In this case only a partial human DNA profile was recovered, and DNA of the person who was bitten was not available for comparison. In field case 2, a police dog was known to have bitten a suspect. A mixed STR DNA profile from at least two sources was identified in which the major component was consistent with the STR DNA profile of the suspect at the 19 STR locus. This DNA profile was available through the Combined DNA Index System (CODIS). The probability of a random match (selecting an unrelated individual at random with a consistent profile) at this locus is 1 in at least 1.7 septillion. Based on the history of no previous contacts between the humans and the dogs, and known bite wounds that drew blood prior to sample collection from the teeth, it is assumed that the human DNA source in both field cases was blood.

**Table 1:** Human DNA profile data derived from technique development scenarios and field cases

		Human DNA Source	Canine Retrieval Site and Time Delay			STR PCR Human DNA Result*	DNA Quantification (ng/µl)	Random Match Probability
Technique Development	Scenario 1	Skin	Canine 1	Muzzle/Hair	Within 2 min	Partial	0.0007	N/A
			Canine 1	Mouth/Teeth	Within 2 min	Partial	0.0006	N/A
			Canine 2	Muzzle/Hair	15 min	Partial	0.0004	N/A
			Canine 2	Mouth/Teeth	15 min	Partial	0.0005	N/A
	Scenario 2	Saliva	Canine 3	Muzzle/Hair	Within 2 min	Consistent	0.0021	~ 4.3e15 (stats at 11 loci)
			Canine 4	Muzzle/Hair	Within 2 min	Consistent	0.0050	~ 3.4e18 (stats at 15 loci)
			Canine 3	Mouth/Teeth	Within 2 min	Match	0.1367	~ 2.5e30 (stats at 23 loci)
			Canine 4	Mouth/Teeth	Within 2 min	Match	0.1354	~ 3.6e30 (stats at 23 loci)
			Canine 3	Mouth/Teeth	15 min	Match	0.0100	~ 2.5e30 (stats at 23 loci)
Field Cases	Case 1	Presumptive Blood	Canine 5	Mouth/Teeth	48 h	Partial	0.0024	N/A
			Canine 6	Mouth/Teeth	48 h	Partial	0.0011	N/A
	Case 2	Presumptive Blood	Canine 7	Mouth/Teeth	6 h	Consistent	0.0214	~ 1.7e24 (stats at 19 loci)

^*^Partial: Identified human DNA. **Consistent**: Identified human DNA profile with one or more inconclusive loci. Inconclusive loci are defined as possible DNA alleles present below the analytical threshold (60 rfu). **Match**: Identified full human DNA profile that matches human control or suspect profile with no inconclusive loci

## Discussion

Police dogs in close contact with suspects may pick up trace human DNA useful in criminal investigations. In this report the feasibility of collecting trace human DNA from canine muzzle hair and teeth was investigated using a swabbing technique, where the DNA source would be human skin, saliva, or presumptive blood. From field case 2, one in which a police dog was known to have bitten a suspect, DNA extracted from swabs of the lower canine teeth produced mixed STR DNA profiles consistent with the suspect’s DNA. In our technique development scenarios we were unsuccessful in retrieving sufficient quality DNA to make a match using the described human touch and swab technique, but partial human DNA was retrieved. Saliva samples had much better results, with consistent DNA when swabs were taken from hair over the muzzle, and matches to the control human DNA when swabs were taken from the mouth. Even repeating sample collection from the mouth after a 15 min delay resulted in a match when the DNA source was saliva. In the authors experience, touch samples tend to provide less complete and poorer quality DNA than that obtained from saliva or blood. Our control subjects were willing to provide saliva samples but not blood, and in our view saliva was comparable to blood for the purposes of this proof-of-concept study. It is worth emphasizing that in the saliva scenario canine unit police officers, who were shown how to collect samples from the hair and mouth of their dogs, provided the samples for technique development, scenario 2. Based on this preliminary data, successful police officer sample collection is feasible, and further comparative studies to establish recommendations that include optimal conditions for DNA collection from dogs by canine unit police officers are warranted.

Police dogs often come into contact with suspects ahead of their handlers, and the contact may include touching or transfer of fluids. Previous reports have shown that touch DNA can be obtained from numerous surfaces [9], including animal fur [10] and feathers [11] in experimental conditions via swab and minitape techniques. In this report, we demonstrate successful recovery of human DNA from canine mouths that matched to control DNA, and that was consistent with a suspect’s DNA. Retrieval of DNA from human-canine interactions can provide valuable evidence in instances when a suspect who a dog reaches escapes arrest. Without a good witness or other means to identify a suspect, trace DNA lifted from the police dog opens the possibility of a match in CODIS, aiding investigations and increasing the likelihood of a positive identification. Consideration should be expanded to include DNA collection from companion animals identified at crime scenes.

## Study limitations

The aim of this report was to establish proof of the concept that a suspect’s DNA could be recovered from police dogs in sufficient quantity and quality to be used in legal investigations. Additional work is needed to both validate the methods described, and to optimize them. In this study there was limited data generated, so no statistical analyses could be derived on, for example, the outcome of variability in swabbing location or technique, variability due to DNA source, or the effects of volume or time on quantity or quality of the DNA retrieved. DNA samples in this study were processed and analyzed by the Arizona Department of Public Safety and the Phoenix Police Department under their forensic protocols. While this ensured a reliable result, it limited the number of, and speed with which samples could be examined. While this created variability in sample storage time between collection and processing, this variance did not appear to negatively impact results and may warrant further investigation.

This study has shown that trace human DNA is retrievable from canine teeth and hair, and may be sufficient to allow consistent or matched results to a suspect. It has also shown that with minimal training police officers can easily collect such samples. Non-suspect DNA in police dog swabs may come from their police handlers or other persons they contact, from the environment, or from the swabs themselves. There is little information available about non-host DNA collected from the oral cavity, and there is no data on if and for how long human DNA would be identifiable in the canine oral cavity. Potential effects of canine salivary enzymes and extracellular nucleic acids, eating, drinking, licking or swallowing, and oral commensal bacteria on the quality and quantity of retrievable DNA are unknown. Results of a second oral swab at 15 min post touch and application of saliva in scenarios 1 and 2 (Canines 2 and 3) was meant to inform planning for future studies related to collection time periods. Lack of re-application of saliva DNA for the 15 min time period in scenario 2 did not appear to change DNA results, but may have reduced the quantity of DNA available on the collection sites. Collection scenarios using longer collection time points would allow a more rigorous evaluation of the swabbing technique and provide collection parameters that would increase the likelihood of a positive outcome.

## Key points

1. Police dogs known or believed to have bitten a suspect can be a source of the suspect’s DNA.
2. Trace human DNA of a quality sufficient to match a suspect can be collected from the hair or teeth of a dog.
3. The collection of trace DNA from police dogs is simple, inexpensive, and is something that trained police officers can easily do.
